# The Role of the Cell Integrity Pathway in Septum Assembly in Yeast

**DOI:** 10.3390/jof7090729

**Published:** 2021-09-06

**Authors:** Cesar Roncero, Rubén Celador, Noelia Sánchez, Patricia García, Yolanda Sánchez

**Affiliations:** Departamento de Microbiología y Genética, Instituto de Biología Funcional y Genómica, CSIC/Universidad de Salamanca, C/Zacarías González, s/n, 37007 Salamanca, Spain; rubencelador@usal.es (R.C.); nsn@usal.es (N.S.); pgr@usal.es (P.G.)

**Keywords:** yeast, cytokinesis, actomyosin ring, septum, cell integrity

## Abstract

Cytokinesis divides a mother cell into two daughter cells at the end of each cell cycle and proceeds via the assembly and constriction of a contractile actomyosin ring (CAR). Ring constriction promotes division furrow ingression, after sister chromatids are segregated to opposing sides of the cleavage plane. Cytokinesis contributes to genome integrity because the cells that fail to complete cytokinesis often reduplicate their chromosomes. While in animal cells, the last steps of cytokinesis involve extracellular matrix remodelling and mid-body abscission, in yeast, CAR constriction is coupled to the synthesis of a polysaccharide septum. To preserve cell integrity during cytokinesis, fungal cells remodel their cell wall through signalling pathways that connect receptors to downstream effectors, initiating a cascade of biological signals. One of the best-studied signalling pathways is the cell wall integrity pathway (CWI) of the budding yeast *Saccharomyces cerevisiae* and its counterpart in the fission yeast *Schizosaccharomyces pombe*, the cell integrity pathway (CIP). Both are signal transduction pathways relying upon a cascade of MAP kinases. However, despite strong similarities in the assembly of the septa in both yeasts, there are significant mechanistic differences, including the relationship of this process with the cell integrity signalling pathways.

## 1. Maintaining the Shape: The Cell Integrity Signaling Pathways

**Figure 1:**
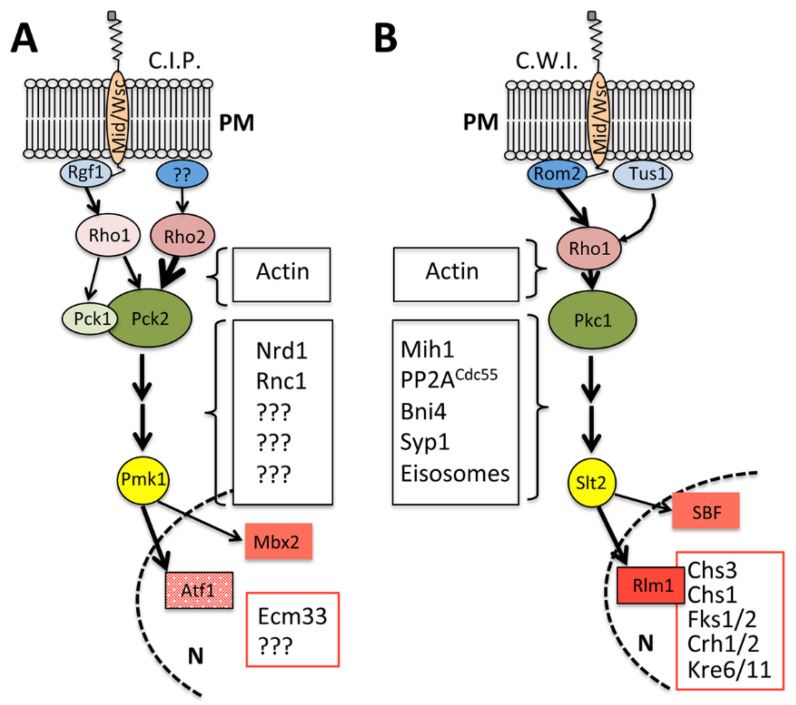
The cell integrity cascade in yeast. (**A**) The cell integrity pathway (CIP) in *Schizosaccharomyces pombe*. (**B**) The cell wall integrity pathway (CWI) in *Saccharomyces cerevisiae*. Both cascades signal from the mechanosensors in the PM (Mid/Wsc) to the transcription factor in the nucleus (N). Red boxed proteins are those for which expression is transcriptionally regulated by the cascade and are involved in the cell wall and/or septum assembly. Black boxed proteins represent cytosolic targets of the cascade at different levels that are related to septum assembly. For additional description of the cascade, see text.

The cell integrity signalling pathways are usually described as fairly linear, they channel the signal from the cell surface to the nucleus without significant branching (Figure 1). Cell wall stress is detected by two conserved families of single-pass transmembrane cell wall sensors of the WSC and MID types. Their role is well documented in *S. cerevisiae*, where these sensors can detect the mechanical tension between the cell wall (CW) and the plasma membrane (PM) [1,2]. They function as upstream triggers of the cell integrity pathway and activate membrane-associated RhoA type GTPases through specific GEFs, the ScRom2p and SpRgf1p [3,4]. In *S. cerevisiae*, signalling is also associated with Tus1p, another Rho1p-GEF and a functional homologue of SpRgf3p, whose relevance in CIP activation is unknown. Signalling in *S. cerevisiae* is directly translated to the MAP kinase cascade through the GTPase Rho1p and its effector the Pkc1p kinase (Figure 1B). In *S. pombe*, the situation is a bit more complex (Figure 1A), there are two different Rho A homologues (Rho1p and Rho2p) and two Pkc kinases (Pck1p and Pck2p), participating in signalling. The major signal input is channelled through Pkc2p, which receives different inputs, the main one through the Rho2p GTPase and a minor one through Rho1p, which also transmits a minor signal through the Pkc1p branch [5,6]. In both yeasts, the signal from the PKC kinase is transmitted to a MAP kinase cascade that ends with the MAP kinases ScSlt2p and SpPmk1p. These kinases participate in phosphorylation events of specific nuclear transcription factors. The *S. pombe* Pmk1p phosphorylates Atf1p and Mbx2p transcription factors, but to date, only a few downstream targets have been characterised. In *S. cerevisiae*, the MAP kinase Slt2p phosphorylates the Rlm1p transcription factor that mediates a strong transcriptional response. This response encompasses genes involved in chitin and glucan synthesis as well as genes encoding cell wall remodelling activities, among others [7,8]. ScSlt2p also phosphorylates the heterodimeric SBF transcription factor that promotes the G1/S transition [9].

In addition to their signalling through transcriptional regulation, these pathways act through cytosolic targets. The Rho1/2p GTPases are directly involved in the biological cycles of actin, regulating patches and filaments turnover that affect endocytosis, cell polarisation and therefore the assembly of the yeast cell wall. Additional targets of the signal transduction in *S. pombe* have been elusive, but are numerous in *S. cerevisiae*. The potential effect of such targets will be described later in the context of septum assembly.

## 2. *S. pombe*, the Fission Yeast

In *S. pombe*, the cells are cylindrical and grow by elongation at their tips. Cell division is accomplished by medial fission using a contractile actomyosin ring (CAR), which guides the formation of the cell wall septum. Cell separation also involves cell wall degradation between the two halves of the division septum that will constitute the new ends of the daughter cells. The overall process has been deeply studied, and there are excellent reviews on cytokinesis [10,11,12,13], septation [14,15] and cell separation [16]. In this review, we will first summarise key steps of fission yeast cytokinesis, including ring assembly, constriction and septum formation, before discussing emerging mechanisms that involve the cell integrity pathway (CIP) in the regulation of cytokinesis and their biological implications.

### 2.1. Before Septum Assembly: Actomyosin Ring Positioning and Assembly

**Figure 2:**
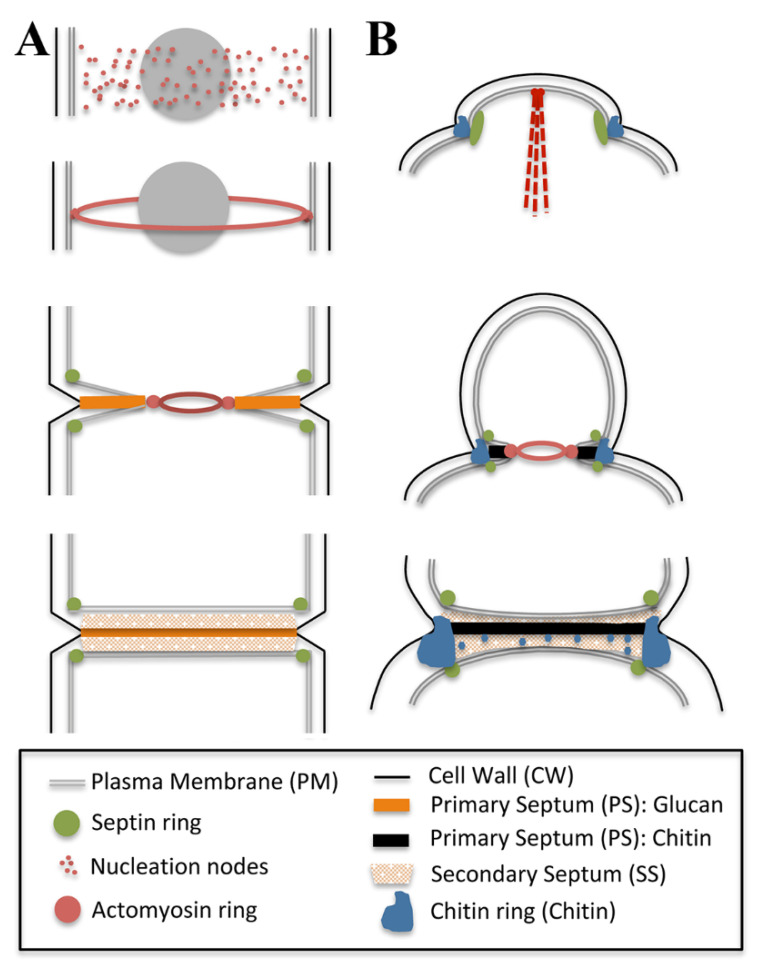
Septum assembly in yeasts: *S. pombe* (**A**) and *S. cerevisiae* (**B**). The schemes represent the temporal sequence of the process, from the early stages involved in the selection of the septation site (upper panels) to the assembly of the secondary septa (lower panels). The final stages in cell separation are not depicted. For additional details in the process, see text.

The CAR in *S. pombe* is composed of short actin filaments assembled by formins and myosin motors (actomyosin) that produce the force to arrange the actin filaments (revised in [12,17]). CAR dynamics are tightly regulated in space and time and can be divided into several steps including positioning, assembly, maintenance, constriction and disassembly (Figure 2A, upper panel). Ring position is determined during interphase by a broad band of cortical cytokinetic precursor nodes located at the equator of the cell [18].

The anillin Mid1p and the DYRK kinase Pom1p dictate CAR positioning in the cell middle [13]. At mitosis, Plo1p kinase activate Mid1p allowing its exit from the nucleus [19,20] and Pak1p kinase promotes its association to the cortical nodes [21]. Then, Mid1p initiates the recruitment of cytokinetic factors, including the IQGAP Rngp2, Myosin II heavy and light chains, the F-BAR protein Cdc15p and the formin Cdc12p (Figure 2A) [13,22].

After spindle pole bodies (SPBs) separation, the cytokinesis nodes condense, and the contractile ring is formed through dynamic interactions between the actin filaments assembled by Cdc12p and Myo2p in adjacent nodes. Afterwards, the ring is maintained until the completion of anaphase in an interval known as maturation that lasts ~10 min until the onset of ring constriction. In maturation, more proteins are recruited from the cytoplasmic pool, while others leave the ring. Mid1p disappears, and the ring adds more polymerised actin, Cdc15p with its partners (Imp2p, Pxl1p, Fic1p and Rgf3p) [23], unconventional myosin-II (Myp2p) [24] and the glucan synthases Bgs1p and Bgs4p [25,26] among others. In the mature ring, proteins are ordered roughly in a three-layered structure. Starting from the inside, the first layer contains membrane-bound proteins that anchor the ring and act as scaffolds, an intermediate layer that contains signalling components that influence cell division and a distal layer that contains F-actin filaments, myosin motors and F-actin cross-linkers [27].

### 2.2. Anchoring the Ring to the Plasma Membrane and the Growing Septum

To perform its function, the CAR needs to be anchored to the PM, a linkage defined by at least three types of attachments: protein–protein and protein–lipid interactions, the cell wall and the arrangement of microtubules at the cell equator.

Upon mitotic entry, more Mid1p binds to the PM, anchoring ring proteins (and then the ring itself) to this structure. It has been shown that Mid1p dimerisation favours its interaction with membrane phospholipids, in particular phosphatidylinositol 4,5-bisphosphate [PI(4,5)P2] [28], preventing the sliding of the CAR [29]. An additional link of the CAR to the PM is provided by Cdc15p (*S. cerevisiae* Hof1p), a CAR scaffold that binds to the membrane through its BAR domain. When *cdc15* expression is repressed, the CAR slides along the PM and disassembles [30,31]. Cdc15p helps to deliver Bgs1p to the PM [30] and binds to paxillin, Pxl1p, Fic1p, Rgf3p (Rho1p-GEF) that also play a role in CAR anchoring [23]. Pxl1p mediates the interaction between the β-glucan synthase Bgs1p and the contractile ring contributing to the initiation of septum synthesis [32].

A different alteration in the cell wall structure also leads to CAR sliding. This has been shown in spherical protoplasts deprived of the wall [33], as well as in cells depleted for Bgs4p that bear low levels of branched-β(1,3)glucan [34], suggesting an additional level of linkage between the CAR and the cell wall. Finally, certain evidence also links the CAR and the cytoskeleton. For instance, microtubule depolymerisation in the β-GS mutant *cps1-191*, which arrests with a stable CAR, leads to CAR sliding [35], and in the absence of the microtubule nucleator Mto2p, the cells also fail to anchor of the CAR in the medial region under conditions that mildly perturb actin structures [36].

### 2.3. Triggering Septation: The Role of the Septation Initiation Network (SIN) in CAR Maintenance and Constriction

Once the CAR is assembled at the division site, it must constrict to guide and power membrane ingression and cell wall synthesis (Figure 2A). The regulation of CAR assembly/maintenance and constriction coupled to septum synthesis depends on a signalling cascade of the septation initiation network (SIN) [37,38,39]. The SIN induces cytokinesis only after the decrease in CDK activity in anaphase, guaranteeing that cytokinesis occurs after chromosome segregation. A pathway similar to the SIN, termed the mitotic exit network (MEN), exists in *S. cerevisiae* [40]. In addition, SIN/MEN orthologues also exist in mammals that conform to the HIPPO pathway [41]. In yeasts, these networks monitor the position of the spindle pole bodies (SPBs), the yeasts equivalent to centrosomes, throughout the cell cycle to coordinate cytokinesis with other cell cycle phases.

The SIN signal begins with the activity of the GTPase Spg1p and involves a regulatory GAP complex, a scaffold complex that anchors the pathway to the SPBs and a linear cascade of three kinases (Cdc7p, Sid1p and Sid2p), in order of their activation [39]. Insufficient SIN results in improper assembly of the contractile ring and failure of cytokinesis, generating multinucleated cells without rings or septa [42]. On the opposite, ectopic activation of the SIN triggers formation of a contractile ring and septum at any point in the cell cycle [43].

How does the SIN achieve CAR assembly and maintenance? What are the SIN substrates that direct CAR constriction and septum synthesis? Most of these questions are still not solved. Among the SIN components, only Sid2p kinase (a nuclear Dbf2-related (NDR) kinase) and its counterpart Mob1p associate with the CAR in mid-late anaphase [44]. Sid2p phosphorylation of Clp1p (*S. cerevisiae* Cdc14p) keeps the phosphatase out of the nucleolus, allowing the protein to operate on cytoplasmic targets. Clp1p reverses Cdk1p phosphorylation of itself, Cdc25p and other Cdk1p substrates antagonising CDK [45]. Clp1p also dephosphorylates Cdc15p, inducing its oligomerisation and the scaffolding activity necessary to recruit CAR components [46]. In addition, Sid2p targets CAR components such as the essential formin Cdc12p, triggering an oligomeric switch that positively modulates formin function [47]. The SIN pathway directly targets the SAD kinase Cdr2p, promoting its dissociation from the cortex [48]. Similarly, Sid2p phosphorylation of Mid1p disrupts Mid1p interaction with membrane [49]. In both situations, removal of these landmark proteins from the cortex cytokinesis resets the division plane for the next cell cycle.

Finally, the SIN should coordinate many aspects of CAR and septum regulation during late cytokinesis, although the molecular mechanism of this regulation remains unexplored. It has been shown that upregulation of the GTPase Rho1p partially rescues the lethality of *sid2* mutants at a low-restrictive temperature [50,51]. Based on these results, it has been proposed that the SIN activates Rho1p, which in turn activates the Bgs enzymes. However, the SIN target(s) involved in septum assembly remain unknown. Therefore, identifying SIN targets [52] and elucidating the consequence of known phosphorylation events at the ring will be central tasks in advancing our understanding of *S. pombe* cytokinesis.

### 2.4. Furrow Ingression and Septum Deposition

Initiation of furrow ingression by *S. pombe* depends on an intact CAR, a signal from the cell cycle clock and septum synthesis (Figure 2A). CAR constriction provides the pulling force for membrane and primary septum deposition, although septum synthesis also contributes to the membrane ingression [17]. The actin and myosin-dependent forces from the ring promote septum deposition and maintain the circularity of the pore [53,54]; in that sense, mutations compromising contractility slow furrow ingression, suggesting that the ring may positively modulate the rate of ingression [24,55]. One of the problems of the CAR being the principal force of ingression is the huge turgor pressure inside the cell that forces the ring to work against this pressure [56]. In addition, furrow ingression and septum growth can still occur in the absence of F-actin. These findings suggest that cell-wall assembly pushing from the outside of the membrane could afford the force for furrow ingression [57]. Moreover, the delivery of exocytic vesicles and membrane edge expansion [58,59,60] could provide stream-like forces, as has been shown in animal and plant cytokinesis [61,62]. A conciliatory model for septum synthesis proposes a two-phase motion. In the first phase, the septum ingresses slowly, remains immature and depends on the CAR integrity; in the second phase, the ingression rate increases, and the CAR becomes dispensable [63].

The fission yeast septum is made of β and α-glucans and lacks chitin. Under electron microscopy, it looks like a three-layered structure with a primary septum (PS) in the middle of two secondary septa (SS). The PS is mainly composed of linear-β(1,3)glucan synthesised by glucan synthase Bgs1p/Cps1p and contains branched-β(1,3)glucan [64,65]. This linear-β(1,3)glucan would play a role similar to chitin in budding yeasts (see below and Figure 2B) and other fungi in the synthesis of the PS. The secondary septum (SS) forms the cell wall once the cells are separated and consists of 1,6 branched β-1,3-glucans synthesised by Bgs4p, α-1,3-glucans synthesised by Ags1p and β-1,6-glucans [15].

One of the most studied aspects of septum synthesis at the PM is the regulation and deposition of the β-glucans. The enzyme responsible is the β–GS complex that consists of four catalytic subunits, Bgs1p-4p, and a unique regulatory subunit, the GTPase Rho1p [66]. Bgs1p, Bgs3p and Bgs4p are essential transmembrane proteins. Bgs1p localises as a ring, tightly associated with the CAR, at the edge of the septum membrane (PM) during ingression and is responsible for the synthesis of the PS. Bgs3p and Bgs4p follow the CAR but remain localised as a disk along the invaginated PM and are required for the synthesis and assembly of the SS [66].

Rho1p activates all three β-glucan synthases and is positively regulated by three guanine nucleotide exchange factors (GEFs): Rgf1p, Rgf2p and Rgf3p [67,68,69]. Rgf3p-GFP is a CAR component and is the main candidate to regulate Rho1p function in septum synthesis. Moreover, *rgf3* depleted cells lyse as couples during cell separation, mimicking the characteristic phenotype of *rho1* depleted mutants and mutants of Pck1p and Pck2p (*S. cerevisiae* Pkc1p homologues). In addition, it is possible that Rgf3p acts as a physical link between components of the CAR and the membrane–bound Bgs-mediated septum growth [27]. CAR-localised proteins, such as Cdc15p, Imp2p and Art1p, recruit Rgf3p, probably activating the regulatory subunit of the β-GS [70,71]. As mentioned before, Cdc15p participates in the transport of Bgs1p to the septum membrane and Rga7p (a Rho GAP) also contributes to the transfer of the Bgs4p to the same area [25,30]; therefore, it is possible that the concerted action of these proteins also regulate traffic of the Bgs1p to the PM. To date, it remains unclear whether Rgf3p regulates only Bgs1p activity or if it is involved in the regulation of all β-glucan synthases.

By contrast, Rgf1p follows the ring from outside, leaving behind a trail as division proceeds [72], making it a likely candidate for regulating the β-glucan synthesis forming the SS. Interestingly, Rgf1p is the most abundant GEF and activates the cell integrity pathway (CIP) in response to cell wall damage and osmotic stress [73,74].

The enzyme responsible for the synthesis of α–glucans in the SS septum is Ags1p, whose activity is regulated by the GTPase Rho2p. Ags1p localization to the CAR is very similar to that of Bgs4p and together with Bgs4p grants to the PS the robustness needed to counteract the turgor pressure for a gradual cell separation [66].

In *S. pombe*, the septin ring apparently does not have a function to recruit proteins for the assembly of the CAR, as occurs in *S. cerevisiae* (see below). This difference is probably associated with the mechanism used by each yeast for the selection of septum position. *S. pombe* septins form a ring structure at the septum during the constriction of the CAR, which serves as a scaffold to recruit the GTPases and glucanases that ultimately mediate daughter cell separation [16]. The glucanases are funnelled by the exocyst and concentrated by the double septin ring. Cell wall degradation starts with the erosion of the wall material that surrounds the septum driven by the α-1,3 glucanase activity of Agn1p [75,76]. After that, cell turgor pressure and the action of the endoglucanase Eng1p [77] finish the dissolution of the PS. Interestingly, the lysis phenotype characteristic of the *rgf3* (*ehs2-1*) mutants is suppressed by elimination of Eng1p but not Agn1p (unpublished results). This result suggests that Rgf3p is involved in PS synthesis and that a balance between cell wall synthesis and degradation is necessary to accomplish cell separation safely.

### 2.5. Integrating Septum Assembly and the Cell Integrity Pathway during Cytokinesis in Fission Yeast

What is the role of the CIP in cytokinesis? Are there cytoplasmic targets of Pmk1p with a potential role in cytokinesis?

In *S. pombe*, the cell integrity pathway (CIP) is composed of a module of three MAP kinases, the MAPKKK Pek1p, the MAPKK Mkh1p and the MAPK Pmk1p, which are regulated by upstream activators, as Rho GTPases and PKC homologues (Figure 1A) [4]. In general, the CIP null mutants display lysis and multiseptated phenotypes characteristic of cell separation defects; however, there are significant differences between mutants in the upper part of the pathways from others in the lower parts. We have described before how Rho1/2p and Pck1/2p regulate the synthesis of the main components of the cell wall, thereby organising the synthesis and/or assembly of the primary and secondary septa. Not surprisingly, most of these mutants shrink as doublets during cytokinesis. In addition, cells of *rgf1*, *pck1* and *pck2* null mutants show monopolar growth [68,78]; suggesting problems in the recognition of a faulty disassembled end [79], a factor that could also affect septum assembly.

Mutants lacking Mkh1p, Skh1p/Pek1p and Pmk1p show separation defects when grown in nutrient-limiting conditions, at high temperature and in hyperosmotic medium [80,81,82]. Apparently, these cells had almost finished cytokinesis but had not completely lysed the external wall, suggesting late defects in cell wall remodelling. Specifically, the phenotype of Pmk1p mutants may reflect fine-tuning of septation, as expected for mutations in CIP repairers of cell wall damage caused under environmental stresses. The differences in the phenotypic penetrance of the different mutants resembled that observed in *S. cerevisiae*.

### 2.6. The Role of the CIP beyond Septum Assembly

In *S. pombe*, contrary to *S. cerevisiae*, the transcriptional response mediated by the CIP seemed rather weak. Upon activation of the route, Pmk1p phosphorylates at least two transcription factors: Atf1p and Mbx2p. Atf1p is also phosphorylated by the SAP MAPK Sty1p in response to various stresses [83]. Atf1p mutants show sensitivity to antifungal agents and the *vic* (*v*iable in the presence of *i*mmunosuppressant and *c*hloride ion) phenotype characteristic of null mutants in bonafide components of the CIP [84,85]. Mbx2p is the closest homologue to Rlm1p in fission yeasts; *mbx*Δ mutants displayed only a modest sensitivity to cell wall-damaging agents, suggesting that Mbx2p plays a minor role in this process. While in *S. cerevisiae*, it is known that Rlm1p regulates the expression of tens of genes, most of which have been implicated in cell wall biogenesis [86], in *S. pombe*, the only well-known target of Atf1p and Mbx2p is Ecm33p. Ecm33p is a glycosyl-phosphatidylinositol (GPI)-anchored cell surface protein; Ecm33p deletion mutants display abnormal morphology and hypersensitivity to antifungal agents, although the relationship between the CIP, Ecm33 and cell wall assembly remains uncertain.

Pmk1p phosphorylation varies periodically during the cell cycle, reaching its maximum activity during cytokinesis. In fact, 15–20% of a population of *pmk1*Δ synchronised cells were unable to complete cytokinesis, suggesting that the Pmk1p pathway is activated to control septum formation and/or dissolution [87]. However, to date, we do not know how this is accomplished. Pmk1p localises to the mitotic spindle and the septum during cytokinesis and constitutively resides in both cytoplasm and the nucleus [88]; however, the role of Pmk1p in cell separation seems mostly independent of its nuclear localisation [89]. Among the few known cytoplasmic targets for Pmk1p in vivo are the RNA-binding proteins Nrd1p and Rnc1p. Nrd1p binds and stabilises the essential myosin II light chain mRNA, thereby playing an important role in the regulation of CAR synthesis and contraction [90]. The potential participation of Rnc1p is unknown.

It has been known for a long time that the MAP kinase Pmk1p becomes activated within minutes by cell wall stress [5,87]; however, it is still unclear how the CIP integrates this stress input with successful cell separation [91]. A quick response is required when the cell’s genome has already split, and the cell becomes ready to separate its cytoplasm. Recently, it has been shown that cell wall damage inflicted during cytokinesis triggers a checkpoint-like response, promoting a delay right before CAR constriction [72]. This delay depends on Rgf1p/Rho1p and Pck2p and was also abolished in the absence of the MAP kinase of the CIP. Because inactivation of this pathway in stressed cells causes defects in septation [80,81,82], it is possible that the CIP signalling delays CAR constriction in response to cell wall perturbations to ensure that cytokinesis reaches completion only after the cell has adjusted to the new conditions.

Finally, there is a connection between the checkpoint response to cell wall damage and the SIN [72]. It has been shown that the cell wall cytokinesis checkpoint depends on the SIN to be achieved. Moreover, the cell wall delay correlates with a prolonged SIN signal. Given that Sid2p is required for CAR maintenance when the cytokinesis checkpoint is active [50], it is very likely that the prolonged SIN activity serves to maintain the CAR in a competent state to achieve constriction safely.

## 3. *S. cerevisiae*, the Budding Yeast

In *S. cerevisiae*, the building of a septum is initiated very early in the cell cycle. Bud site selection is mediated by the landmark proteins inherited during cell division [92]. Then a cascade of GTP-GDP-bound proteins recruits and activates the Rho family GTPase Cdc42p, which in turn stimulates actin cable polarisation, targeted exocytosis and septin ring formation [93]. Here, we shall focus on the assembly of the septin ring that in *S. cerevisiae* acts as a scaffold structure for the sequential recruitment of the components that build up a septum.

The initial septin ring recruits the major chitin synthase, Chs3p, and its activator Chs4p, to the mother site of the neck. There, they will promote the synthesis of the chitin ring that serves as a scaffold for the septum, although it is not strictly a part of it. The initial septin ring undergoes significant structural modifications monitored by the morphogenesis checkpoint that finally result in the split of the septin hourglass structure into a double ring. Formation of this structure is not essential for cytokinesis, but it could set the limits of the area for septum assembly [94], favoring the correct positioning of the ingression progression complexes (IPCs) to trigger the synthesis of the primary septum. In addition, the double septin ring acts as a landmark to redirect the polarised secretion in the daughter cell to the neck region, facilitating the assembly of the septum [95,96].

### 3.1. Before Septum Assembly: Shaping the Yeast Cell through the Synthesis and Assembly of the Cell Wall

Although this review is mainly focused on the role of CWI in septum assembly, we could not ignore the general roles of CWI in the synthesis of the yeast cell wall. Activation of Cdc42p at the site of bud formation triggers the synthesis of new cellular material forming the growing bud (Figure 2B) [97]. This material includes β-glucans synthesised by the β-glucan synthases Fks1p and Fks2p. The FKS1/2 are activated by the Rho1p GTPase [98,99] and its GEF Rom2p recruited to the site of bud emergence by the CWI sensors, Mid2p and Wsc1p [100]. At the same time, the main CWI Kinase PKC1 is recruited to the site of polarised growth [101], allowing the synthesis of the major component of the yeast cell wall. Later on, the polarisation machinery is displaced to the growing tip (Figure 2B, upper panel), where the synthesis of the cell wall continues along the cell cycle. Interestingly, it has been elegantly shown that the CWI is engaged in the localisation of the cellular machinery after the local cell wall is damaged through proteasomal degradation of critical components previously assembled at the site of cell division [102].

The structure of the septin ring is externally reinforced by a chitin ring synthesized by Chs3p, defining the width of the yeast neck [103]. The absence of the chitin ring, which is not essential on its own, exacerbates minor cytokinesis defects leading to severe synthetic lethal effects [104,105]; moreover, upregulation of the chitin synthesis mediated by Chs3p relieves multiple strong cell wall defects mediated by antifungal therapies [106]. In this scenario, it is therefore, not surprising to find chitin synthesis under the control of the CWI. How is this control achieved?

It has been shown that phosphorylation of Chs3p depends on PKC1 activity [107], although there is no direct evidence that the CWI activates chitin synthesis. However, there are multiple circumstantial pieces of evidence indicating that this could be indeed the case. Chs3p activity depends on its polarised delivery to the neck where it binds to the septins [108]. This localisation strictly depends on the endocytic turnover of Chs3p [109,110]; therefore, the potential effects of the CWI on actin patch localisation (see below) will affect Chs3p endocytosis. This effect can be direct or could be mediated through Chs4p that links Chs3p to the septins [109,111]. A more important action of CWI in chitin synthesis is through the transcriptional regulation of the *GFA1* gene encoding one of the enzymes required for the synthesis of the UDP N-acetylglucosamine (UDP-NAGA), the metabolic precursor of chitin. Gfa1p is normally synthesised in limited amounts and acts as a bottleneck in chitin synthesis [112]. Cell wall damage triggers a compensatory response accompanied by a significant increase in chitin synthesis that relays in higher levels of Gfa1p, promoted by the activation of the CWI response through its transcriptional program [7]. Not surprisingly, overexpression of GFA1 by other means also alleviated many cell wall defects. The regulation of the Chs3p related to the synthesis of chitin in the daughter cell and in the secondary septa, will be describe later.

Finally, a potential role of the CWI in septum assembly through the ER stress surveillance (ERSU) cell cycle checkpoint that ensures that cells inherit functional ER into the daughter cell cannot be ignored; the role of CWI in this process is well documented, but the molecular mechanisms that underlie this function are still poorly defined (reviewed in [113]).

### 3.2. Starting the Separation: The Building of a Primary Septum

While yeast cells progress along the cell cycle, cell growth moves from apical to isodiametric, and the septin ring undergoes strong structural modifications that eventually end with splitting in a double ring that would mark the position of the septa [95,96]. This progression is monitored by the morphogenesis checkpoint through the function of the Swe1p kinase [93].

Progression along the cell cycle triggers the so-called mitotic exit system, which involves the Cdc14p early anaphase release (FEAR) and the mitotic exit network (MEN) which is the start point for cytokinesis. This signal triggers the destruction of the mitotic kinases allowing the arrival of the chitin synthase Chs2p to the septation site [114,115]. In addition, the mitotic exit also promotes the Rho1p-mediated assembly of the actomyosin ring though the Bni1p formin [116,117]. Concomitantly, the symmetric relocalisation of the polarisation machinery to both sites of the division site funnels the secretion machinery in order to provide the building blocks for the synthesis of the new membrane units and the septum. The primary septum (PS) is then assembled (Figure 2B) by the coordinated action of two independent but interconnected mechanisms: the centripetal synthesis of the chitin disk and the contraction of the actomyosin ring [118].

The chitinous nature of the primary septa in *S. cerevisiae* represents a strong difference with *S. pombe* PS, which is mainly made of linear β-(1,3)-glucan [14,64]. In fission yeast, the CWI could control the synthesis of PS by modulating Rho1p and/or Bgs1p, the regulatory and the catalytic subunit, respectively, of the β-glucan synthase complex [119]. In *S. cerevisiae,* the activation of Chs2p orchestrated by the CWI pathway has not been described to date.

The pioneering work in Cabib and Li labs established that chitin synthesis mediated by Chs2p and the actomyosin ring contraction are two interdependent, but interconnected processes that led to the synthesis of the primary septa [120,121]. Chs2p dephosphorylation promoted by the MEN substrate Cdc14p triggers its exit from the ER and its delivery to the division site, which ensures that septum formation takes place only after the completion of mitotic events [114,115]. Then, Chs2p interacts with the SH3 domains of Hof1p (SpCdc15p) as well as with Cyk3p (SpCyk3p), Inn1p (SpFic1p) and with the scaffold Spa2p, favouring its incorporation to the IPC complexes, where is activated to synthesise the chitin disk [122,123]. It has been proposed that the C2 domain of Inn1p participates in Chs2p activation [123]. Accordingly, some hypermorphic alleles of Chs2p can bypass the cell division defect seen in Inn1p mutants and mutants of other IPC components [124]. However, the molecular mechanism of Chs2p activation at the neck remains uncertain.

It is known that the chitin synthases stay competent for chitin synthesis after endocytosis blockade [109]; therefore, modification of the endocytic turnover by the CWI pathway through Rho1p could have a direct impact on PS synthesis [125,126]. In this sense, it has been shown that Rho1p and Pkc1p modulate the neck localisation of Syp1p, a protein involved in the negative regulation of actin patch assembly [127,128]. In addition, activation of the CWI induces phosphorylation of the eisosome core components such as Pil1p and Lsp1p [129], which could participate in PM compartmentalisation [130]. Altogether, this evidence suggests a direct role of CWI in regulating the endocytic turnover of proteins involved in septum assembly. Finally, the MEN kinase Dbf2 directly phosphorylated Chs2p, triggers its dissociation from the neck [131].

### 3.3. At the End of the Process, Secondary Septa Synthesis and Cell Separation

At the end of the PS assembly, there begins the synthesis of the SS, which in *S. cerevisiae* is formed by β-glucans with minor quantities of chitin. Chitin is synthesised by the chitin synthase Chs3p [132], and its localisation at the neck is dependent on Rho1p [133], although the details are unknown. One possibility is that the relocalisation of secretory and endocytic machinery on both sides of the neck increased the endocytic turnover of Chs3p/Chs4p, favouring chitin synthesis at the SS. The regulation of the β-glucan synthesis at the SS by the CWI is achieved through Rho1p. During septation Rho1p is recruited and activated by a distinct mechanism that involves its binding to membrane phosphoinositide’s in order to activate the β-glucan synthases Fks1/2p [133]. In addition, SS synthesis could be also achieved through transcriptional activation of FKS2 and GFA1 genes by the CWI pathway.

Moreover, chitin and β-glucans are linked together at the septa by the action of Chr1/2p transglycosidases, which are localised at the neck [134]. In the neck region, cross-linkage occurs between the β-(1-3)-glucans and the Chs3p-made chitin, therefore linking the SS β-glucan material to the chitin ring [135]. However, additional linkages between chitin and β(1-6) glucans cannot be discarded as part of the SS layered out in the mother cells of the septa, as it has been reported for the lateral cell walls [135]. Interestingly, Chr1/2p expression is under the transcriptional control of the CWI, which could also contribute to the strength of the septa through increased levels of chitin-glucans cross-linkages.

The last step in septum dynamics is its dissolution to achieve cell separation. This process is triggered by the RAM pathway and involves the Ace2p mediated expression of several hydrolytic enzymes, specifically in the daughter cell [136]. In *S. cerevisiae*, the major role in cell separation is performed by the Cts1p chitinase [137] together with the Eng1p endoglucanase [138]. These proteins are secreted in a polarised manner to the periplasmic space surrounding the neck region of the daughter cell [138,139]. Chitinase acts centripetally, first on the chitin ring and later on the chitin disk formed by Chs2p, allowing the separation of chitin from the SS material mainly formed by β-glucans. The partial degradation of β-glucans by Eng1p would contribute to the process. Interestingly, these actions, performed only from the daughter side, leave most of the primary septa at the mother cell as the bud scar.

Cell separation involves degradation coupled to the repair of the cell wall when degradation takes place in an excess that could compromise cell integrity. Therefore, the CWI performs a critical role by regulating the expression of the repair enzyme, Chs1p [7]. Interestingly, while the transcriptional response mediated by the CWI pathway is not relevant under normal circumstances, the transcriptional regulation of Chs1p becomes critical in the presence of an excess of chitinase activity [104,140]. More recently, it has been described a sort of cell separation checkpoint-like named ECO (enforcement of cytokinesis order) that down-regulates directly Cts1p secretion upon cytokinesis defects. The ECO detects cytokinesis defects and signals through the Cbk1p kinase in order to prevent Cts1p secretion independently of the transcriptional regulation exerted by Cbk1p trough the RAM pathway [141]. The precise mechanism used by this pathway is uncertain and, so far, its potential relationship with the CWI is untested.

### 3.4. Beyond Septal Assembly: The Generation of a Remedial Septum

Besides the roles of CWI in septum assembly, the CWI participates in the synthesis of the remedial septa [118]. These septa are assembled upon catastrophic events caused by defects in Chs2p or in the actomyosin and septin rings. The failure in separating mother and daughter cells compromises cell integrity and therefore triggers a strong CWI response. This response is directed in part by the chitin synthase Chs3p, which promotes an abundant synthesis of chitin at the neck region independently of its role in the assembly of the chitin ring. Synthesis is triggered by up-regulation of the *GFA1* gene, although the collapse on the actomyosin ring contraction very likely favoured Chs3p accumulation at the neck by preventing its endocytosis. In addition, FKS1/2 and CHR1/2 genes are also upregulated [7,112], increasing chitin-glucans cross-linkages and in general favouring the strength of the remedial septa. Moreover, the activation of CWI triggers the increased expression of genes involved in the synthesis of β-(1,6)-glucans and mannoproteins that could also contribute to its assembly. Altogether, these actions promote the synthesis of abnormally engrossed septa that lack the typical layered structure but prevent cell lysis during cytokinesis.

As stated above, the upregulation of chitin synthesis mediated by the CWI seemed to be a cellular general response against cell wall damage that can be mimicked simply by the addition of glucosamine to the media as a direct precursor in chitin synthesis [104].

### 3.5. The Action of CWI beyond Septum Assembly

The CWI pathway has been described extensively based on its strong transcriptional in response to cell injuries that compromised cell integrity. Due to the different phenotypic penetrance of mutations in their components, very soon it was apparent that the CWI pathway was not linear. The phenotypes associated with the absence of the Rlm1p transcription factor were milder than those associated with the upper part of the route, while the phenotypes seen in the absence of the Slt2p MAP kinase were clearly intermediate, suggesting significant branching in the functional signalling along the route. We have highlighted before some of the most relevant non-transcriptional effects of the CWI response in the assembly of a functional septum, but many other aspects have not yet been addressed.

In recent years, and through different approaches, it has been shown that the CWI mediates, directly or indirectly, the phosphorylation of multiple proteins, potentially influencing all aspects of cell physiology, including carbohydrate metabolism, protein synthesis and DNA repair, among others [100]. There is also a close relationship between cell wall synthesis and cell cycle progression; therefore, a tight link between CWI and cell cycle progression is expected, exemplified by early reports that established the SBF complex as a direct target of the CWI [142]. This linkage is established at multiple levels that have been reviewed recently [100]. However, we shall highlight here the close relationship between the PP2A^Cdc55^-Zds1/Zds2 complex and the CWI. This PP2A^Cdc55^ complex is an effector of Rho1p that in unperturbed growth conditions favours polarised cell growth, inactivating the Rho1p GAP Lrg1, while preventing CWI activation by stabilising the other Rho1p GAP, Lrg7. Upon cell wall stress, Rho1p rapidly activates the CWI, which downregulates the PP2A^Cdc55^ complex at multiple levels, reducing polarised growth in favour of the stress response [143].

An additional link between the CWI and cell cycle progression has been recently proposed through the protein Bni4p, a direct target of the MAP kinase Slt2p [144] and the cyclin kinase Pho85p [145]. Bni4p is involved in septum assembly [146] and its location at the neck region is cell-cycle-regulated [147], allowing a new level of the crosstalk between CWI and cell cycle progression. It is tempting to speculate a potential functional relationship between this level of control and the new check-point-like response associated with the cell wall damage inflicted during cytokinesis recently described in *S. pombe* [72]. However, the answer will have to wait until the identification of the potential target/s of the CIP at the neck.

## 4. Concluding Remarks and Future Perspectives

In this review, we have highlighted the multiple interconnections between the cell integrity signalling responses and the assembly of yeast septa using two model yeasts with different modes of growth. We believe that though the main rules governing the physiological relationship between cell integrity signalling and septum assembly are similar, sometimes the molecular mechanisms underlying both processes are different.

Some of the differences described in the text may reflect the levels of knowledge accumulated for both yeasts. However, in many others, the differences are probably associated with how both yeasts choose the site of septum synthesis, the different structure and composition of their cell walls and so on. In this context, it would be very interesting to know how the CWI influences septum assembly in other fungi. The CWI response seems conserved across fungi and participates in the response to several stresses, including antifungal therapies and in pathogeny [148]. In addition, although some members of the cascade have been linked with the synthesis of α and β-glucans, the precise implications of this cascade in septum assembly have been poorly explored.

In summary, it would be interesting to gain a deeper understanding of the relationship between CWI and septum assembly in these yeasts as well as in other fungi to define the general rules governing them. This knowledge eventually will allow the identification of new targets useful in the design of efficient antifungal therapies.
